# Total Binding Affinity Profiles of Regulatory Regions Predict Transcription Factor Binding and Gene Expression in Human Cells

**DOI:** 10.1371/journal.pone.0143627

**Published:** 2015-11-24

**Authors:** Elena Grassi, Ettore Zapparoli, Ivan Molineris, Paolo Provero

**Affiliations:** 1 Dept. of Molecular Biotechnology and Health Sciences, University of Turin, Turin, Italy; 2 Center for Translational Genomics and Bioinformatics, San Raffaele Scientific Institute, Milan, Italy; University of Leuven, BELGIUM

## Abstract

Transcription factors regulate gene expression by binding regulatory DNA. Understanding the rules governing such binding is an essential step in describing the network of regulatory interactions, and its pathological alterations. We show that describing regulatory regions in terms of their profile of total binding affinities for transcription factors leads to increased predictive power compared to methods based on the identification of discrete binding sites. This applies both to the prediction of transcription factor binding as revealed by ChIP-seq experiments and to the prediction of gene expression through RNA-seq. Further significant improvements in predictive power are obtained when regulatory regions are defined based on chromatin states inferred from histone modification data.

## Introduction

A major goal of molecular biology in the post-genomic era is to understand the network of regulatory interactions that allow a single genome to produce hundreds of distinct cell types, and to react to external stimuli with the appropriate gene expression response. The most fundamental layer of gene regulation happens at the transcriptional level, where transcription factors (TFs) bind regulatory regions located in the proximity of their target genes and affect the binding of the transcriptional machinery and their level of expression.

The first step in understanding transcriptional regulation is to predict the DNA sequences to which a TF is able to bind, so as to identify its targets. Most TFs bind sequences that are relatively short and degenerate, making this prediction quite challenging. The degeneracy of the binding sites is reflected in the use of a Positional Weight Matrix (PWM) to describe the binding preferences of a TF. A PWM specifies the frequency distribution of the 4 nucleotides in each position of a binding site, and is typically used to assign a score to each DNA sequence. The score expresses the degree of similarity between the observed sequence and the PWM. In the most common approach, a sequence is then predicted to be a transcription factor binding site (TFBS) if it scores above a given cutoff.

The need for a cutoff is unsatisfactory not only because it introduces an arbitrary parameter, but also and especially because recent detailed investigations of transcription factor binding have shown it to be a thermodynamic process in which transient binding to low-affinity sequences plays an important role [1]. In this view the concept itself of a binary distinction between binding and non-binding sites comes into question: It becomes more appropriate to consider the total binding affinity (TBA) of a sequence taking contributions from both high- and low-affinity sites. TBA was introduced and applied to transcriptional regulation in yeast by the Bussemaker lab [2, 3], and weighs binding sites of all possible strengths based on a physical model of TF:DNA interactions.

Recently we used TBA profiles to study the evolution of cis-regulatory regions in humans [4]: indeed TBA profiles are ideal to study the evolution of regulatory networks since they naturally take into account the widespread phenomenon of binding site turnover [5]. Other cutoff-free methods of using PWMs to predict TF binding were proposed in [6–10].

However a systematic investigation on the use of TBA profiles in studying gene regulation is still lacking. Indeed in the studies cited above no systematic attempt was made to predict the functional outcome of TF binding, that is gene expression, using TBA. Moreover, knowledge of chromatin states as provided by histone modification marks was not used to improve the definition of regulatory sequences and thus obtain more accurate TBA profiles.

In this work we fill these gaps and show that TBA profiles of regulatory regions are systematically superior to cutoff-based methods in predicting both TF binding and gene expression in human cells. Including information about chromatin state in the definition of the regulatory regions further improves the predictive power of TBA.

## Methods

### Downloading and associating ChIP-seq data and PWMs

ChIP-seq peaks were downloaded from the hg19 UCSC Track “Transcription Factor ChIP-seq Uniform Peaks from ENCODE/Analysis”. This track represents a collection of all the ChIP-seq experiments performed in human cell lines for the ENCODE project re-analyzed with a uniform pipeline. We obtained 690 files of ChIP-seq peaks performed with 91 human cell lines and representing 161 unique regulatory factors.

To define a wide collection of human PWMs we used the Bioconductor Package MotifDB ([11]), that collects TF binding preference information from several sources (among which Jaspar [12], UniPROBE [13], hPDI [14] and Ref. [15]). We were able to retrieve 1298 matrices associated with a human Entrez ID. Shuffled PWMs were obtained by randomly permuting the positions, thus maintaining the base composition of each PWM.

Entrez IDs were used to link the target of a given antibody used for a ChIP-seq with its corresponding PWM, resulting in 689 peak files associated with at least a PWM. Due to redundancies in the MotifDB collection of matrices and in the ENCODE ChIP-seq antibodies we end up with 992 experiment/PWM pairs, representing 104 different antibodies, 85 Entrez IDs and 203 PWMs.

As negative controls for determining Receiver Operating Characteristic (ROC) curves we used the peaks obtained in HeLa cells with total mouse IgG used in the IP step, obtained from another UCSC track (Yale, that is one of the tracks containing data used for the Uniform one—the negative control experiments were not included in Uniform). To ensure that our results do not depend on this choice of negative controls we performed some tests using as negatives the total input derived peaks and random sequences following the same size distribution of total mouse IgG peaks or of the real ChIP-seq peaks, and the results were always comparable.

### TBA and occupancy

Total affinity is defined as in [4]: *a*_*rw*_ of a PWM *w* for a sequence *r* is given by:

$$\begin{array}{c} a_{rw}=\log\sum_{i=1}^{L-l}\max\left(\prod_{j=1}^{l}\frac{P(w_{j},r_{i+j})}{P(b,r_{i+j})},\prod_{j=1}^{l}\frac{P(w_{l-j+1},r_{i+j}^{\prime })}{P(b,r_{i+j})}\right) \end{array}$$ (1)

where *l* is the length of the PWM *w*, *L* is the length of the sequence *r*, *r*_*i*_ is the nucleotide at the position *i* of the sequence *r* on the plus strand, $r_{i}^{\prime }$ is the nucleotide in the same position but on the other strand, *P*(*w*_*j*_, *r*_*i*_) is the probability to observe the given nucleotide *r*_*i*_ at the position *j* of the PWM *w* and *P*(*b*, *r*_*i*_) is the background probability to observe the same nucleotide *r*_*i*_.

In the usual approach aimed at identifying discrete binding sites one usually defines the score of a sequence of length *l* as

$$\begin{array}{c} S\left(w,r\right)=\log\;\max\left(\prod_{j=1}^{l}\frac{P(w_{j},r_{j})}{P(b,r_{j})},\prod_{j=1}^{l}\frac{P(w_{l-j+1},r_{j}^{\prime })}{P(b,r_{j})}\right) \end{array}$$ (2)

If *S*_*max*_(*w*) is the maximum possible score for the PWM, binding sites are then defined as all sites scoring better than *C* ⋅ *S*_*max*_, with 0 < *C* < 1.

To compare TBA to cutoff-based methods we defined a cutoff-dependent *occupancy* which is computed like the TBA, but limiting the sum to sites scoring better than *C* ⋅ *S*_*max*_:

$$\begin{array}{ccc} t_{rwc} & = & \log\sum_{i=1}^{L-l}\max\left(\prod_{j=1}^{l}\frac{P(w_{j},r_{i+j})}{P(b,r_{i+j})},\prod_{j=1}^{l}\frac{P(w_{l-j+1},r_{i+j}^{\prime })}{P(b,r_{i+j})}\right)\\ & \times & \phi (C,w,r,i) \end{array}$$ (3)

where

$$\begin{array}{c} \phi \left(C,w,r,i\right)=\left\{\begin{array}{cc} 1 & \;\text{if}\;S\left(w,r,i\right)>C\cdot S_{max}\left(w\right)\\ 0 & \;\text{otherwise} \end{array}\right. \end{array}$$ (4)

Therefore the occupancy is computed exactly like the TBA, but ignoring all contribution from sequences scoring less than *C* ⋅ *S*_*max*_. Background bases frequencies were obtained from intergenic regions in the hg19 release of the human genome.

MotifDB PWMs are expressed as probabilities and in some cases are equal to zero: we applied a standard “pseudocount” of (1 × 10^−6^), smaller than the smallest non-zero probability. PWMs directly downloaded from Jaspar CORE have counts, in this case we transformed zeroes to ones before converting counts to probabilities.

#### Affinity and occupancies on human promoters

The TBA and occupancy values (with 10 equally spaced values of the score cutoff *C* from 0.1 to 1) for all PWMs were computed over human proximal promoters, defined as sequences 1500 bases upstream and 500 downstream of the transcription start sites (TSS) as reported in the RefSeq track of the UCSC genome browser (version hg19, February 2009). To focus on genes unambiguosly associated to a single TSS we restricted our analyses to 12,681 genes associated with a single transcript (according to the GENCODE gene ID—Refseq mapping obtained from Ensembl BioMart 73).

### Gene expression

Long PolyA+ RNA-seq cytosolic (for GM12878, H1-hESC, HUVEC, HepG2 and K562 cell lines) or cell extracts (for HMEC, HSMM, NHEK and NHLF cell lines) datasets were downloaded from the ENCODE/Cold Spring Harbor Lab Long RNA-seq track, obtaining expression level estimates at the gene level. We chose the 9 cell lines for which a Hidden Markov Model (HMM) is available to define regulatory regions using chromatin states (see below). Values were normalized by adding a pseudo-count, namely the smallest expression value different from zero, to each value and then log2-transformed.

### Inclusion of chromatin HMMs

15-state HMM datasets were downloaded from the release 1 of Broad ChromHMM UCSC track, converting hg18 genomic coordinates to hg19 via liftover. These chromatin predictions are available for the same nine cell lines for which we downloaded gene expression data. Using this segmentation model we classified the 15 states into 13 “open chromatin” states including promoter, enhancers and transcriptionally active states; and two “closed” states (“Polycomb repressed” and “Heterochrom; low signal” in the terminology of [16]).

We then used these chromatin state predictions to better define our proximal promoters: masking (i.e. not calculating affinity or occupancy values for those subsequences) positions overlapping “closed chromatin” regions and expanding each masked proximal promoter region to the maximal overlapping “open” region. The resulting sequences were used as regulatory regions based on chromatin states.

### AUC and linear models

ROC and AUC analyses were performed using the R ROC package [17]—to compare the predictive powers of 10 different occupancy cutoffs and TBA we calculated 11 AUC values for every experiment/PWM pair: one for the TBA and 10 for equally spaced cutoffs used to define occupancy. As negative cases we used the total murine IgG peaks and as positive ones the Uniform track peaks. The ROC curves were computed, separately for each cutoff value, by using the occupancy value for all positive and negative cases as predictors. The P-values reported in S1 Dataset and associated to each AUC value derive from Mann-Whitney U tests comparing occupancy or TBA values of positive and negative cases. For 18 experiment/PWM pairs the AUC was <0.5 for all values of the cutoff, indicating complete lack of predictive power. This might be due either to problems either in the PWM or the ChIP-seq data. These cases were removed, so that we ended up analyzing 188 PWMs.

To understand if TBA could be used to model gene expression we fitted linear models with gene expression values as the dependent variable and TBA or occupancies as independent ones. Every gene was associated with its expression value (in a specific cell line) and TBA values calculated on its promoter or on its chromatin-defined regulatory regions (as described above). A linear model was then fitted with the R function lm from the stats package. Each linear model is characterized by a coefficient of determination (*R* ^2^), that reflects the fraction of variance in gene expression that can be explained with a linear composition of the TBA values (plus a costant value, the intercept), and a P-value related to the statistical significance of the of the whole model. More precisely, the P-value reflects the statistical significance of the improvement in predictive power obtained by using the TBA values as independent variables compared with a model with intercept only (that is, where gene expression does not depend on TBA values). All the *R* ^2^ values we quote are adjusted for the number of predictors in the model.

In addition to these measures referring to the complete model, multivariate linear regression also yields a coefficient and a significance value for each of the dependent variables (i.e. TBA for different PWMs), reflecting the influence of its values on the independent one (i.e. gene expression) and taking into account the global effect of all the other variables. In this context it is advisable to use a small set of well defined PWMs without redundancies to avoid to set up an overdetermined model and to be able to easily interpret the coefficients—therefore for these models we used a reduced collection of 130 PWMs from Jaspar CORE.

Cross validation and lasso penalized regression where used to test for possible overfitting and redundance of the regressors. We performed a standard 10 fold cross validation and lasso regression respectively with the R packages bootstrap and glmnet [18, 19].

### Software availability

In order to be able to compute total affinities and occupancy values on many sequences in an efficient manner we developed the MatrixRider Bioconductor package [20] that given a (multi)fasta file with the desired sequences and the background frequencies produces either the total affinities or the total occupancies with a given cutoff. PWM data are obtained via the JASPAR2014 [21] or the MotifDB package [11].

## Results

### Predicting transcription factor binding with and without score cutoffs

We set out to determine the effectiveness of TBA in predicting *in vivo* binding of TFs to regulatory regions and to compare it to methods using score cutoffs to define discrete candidate binding sites. We used a large collection of ChIP-seq datasets available from the UCSC genome browser, and associated PWMs to TFs as described in the Methods.

Given a sequence, and a PWM of length *L* we computed the likelihood score of all the subsequences of length *L*. These were used as described in the Methods section to compute the TBA and the *occupancy* of the sequence at various values of the score cutoff. The TBA sums the contributions of all subsequences of the original sequences, weighted by their likelihood ratio, while the occupancy limits the sum to subsequences scoring better than a given cutoff *C*, expressed as a fraction of the maximum possible score.

We then sought to compare the predictive power of the TBA and occupancy at various cutoff values, as measured by the area under the ROC curve (AUC). For each ChIP-seq dataset the bound sequences that we want to predict are given by the peaks provided in the UCSC track. As negative data for all the datasets we used the peaks obtained in Hela cells with total mouse IgG used in the IP step, which are available from the Yale track. We then computed the AUC for the TBA and 10 equally spaced values of the score cutoff *C*, from 0.1 to 1 (taking *C* = 1 means considering only the contribution of the highest scoring sequence).

**Fig 1:**
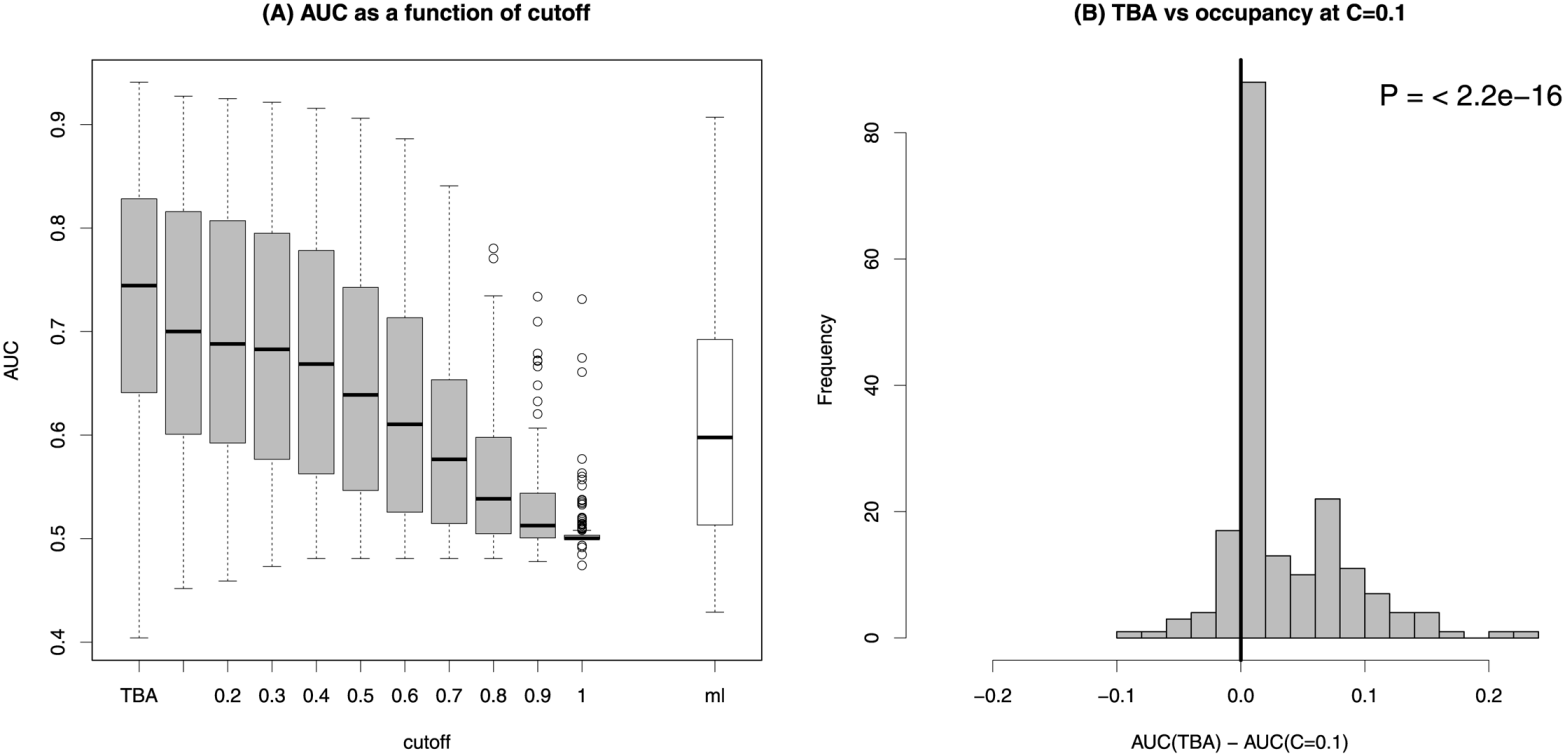
(A) Boxplot of the AUC of 188 PWMs using TBA and occupancy at increasing values of the score cutoff. The white bar shows the distribution of AUCs obtained with MotifLocator [25], using the sum of the scores of the sites above an 80% cutoff as sequence score. (B) Distribution of the difference in AUC between TBA and occupancy at cutoff 0.1 for the same PWMs. The *P*-value is obtained with a paired Wilcoxon test.

The AUC for the 188 PWMs we could analyze, each averaged over all relevant ChIP-seq experiments, are shown together as a boxplot in Fig 1A, which shows that TBA is in general a better predictor of binding than any cutoff-based definition of occupancy. In particular a dramatic decrease in predictive power is observed at the cutoff values of order 0.6∼0.8, that is those that are typically used to identify discrete binding sites by most prediction algorithms (see e.g. [22–25]). However the performance of the TBA is higher than the performance of occupancy at all cutoff values: for *C* = 0.1 the distribution of the difference in AUC between TBA and occupancy for the 188 PWMs is shown in Fig 1B. Complete AUC values are reported in S1 Dataset and represented as a heatmap in S1 Fig.

Note that even with the lowest cutoff we examined (*C* = 0.1) occupancy gets contribution from a rather small portion of the sequence: for example for a PWM of intermediate information content such as the JASPAR Core matrix representing NFI CCAAT-binding (MA0161.1) we find an average of 27 sites per ChIP-seq peak, covering 8.9% of the peaks.

We compared our results with those of another tool (“MotifLocator” [25]) which produces, for a given sequence and a PWM, the list of sites scoring better than a given cutoff. We used the sum of all scores above a cutoff as a score for the whole sequence. With no cutoff we obtained essentially no predictive power. This shows that it is not enough to take weak sites into account: sites of different affinities must be weighted according to a physical model such as the one described in [2], which leads to the definition of TBA. Summing all scores above an 80% cutoff we obtained the AUCs shown by the white bar in Fig 1A.

These results show that binding sites of arbitrarily low affinity contribute to TF binding as revealed by ChIP-seq data. The TBA of a sequence takes into account all binding sites and weighs them based on a physical model of TF:DNA interaction, and turns out to be a better predictor of TF binding than quantities based on the identification of a discrete set of binding sites.

### TBA predicts gene expression

We then asked whether TBA can be used to predict gene expression in human cells, and how its predictive power compares to methods based on discrete binding sites. We used RNA-seq experiments performed within the ENCODE project on 9 human cell lines, and we modeled the dependence of gene expression on TBA profiles with log-linear regression (see [26] for a comparison of various models): the logarithmic expression *e*_*g*_ of gene *g* is given by

$$\begin{array}{c} e_{g}=\sum_{i}c_{i}a_{g}^{\left(i\right)}+b+r_{g} \end{array}$$ (5)

where $a_{g}^{\left(i\right)}$ is the TBA of the regulatory region of *g* for PWM *i*, *c*_*i*_ and *b* are parameters to be fitted and *r*_*g*_ is the residual. The index *i* runs over a suitable collection of PWMs. The model is fitted separately for each cell line, so that the fitted *c*_*i*_’s reflect the relevance of PWM *i* for each cell type (or rather the relevance of the TFs whose binding sites are described by the PWM). This is a radically simplified model based on the following assumptions:

1. the expression *e*_*g*_ is given by a basal level *b* plus a “regulated” term
2. the basal level is the same for all genes, and thus differences in expression between genes are entirely due to differences in TBA profiles of their regulatory regions
3. PWMs contribute additively to *e*_*g*_

The coefficients *c*_*i*_ can be either positive or negative and reflect the relevance of the TFs binding that PWM to gene expression in the cell type being studied. For example we expect the *c*_*i*_ of a transcriptional activator to be positive if the activator is expressed in the cells.

The model has obvious limits, since for example it cannot cope with a TF acting as an activator of some genes and a repressor of other genes; nor can it take into account cooperative effects. A model which circumvents this difficulty can be achieved by using the principal components of the TBA as independent variables (see e.g. [26]). However our goal here is to evaluate the predictive power of the model in terms of the gene expression variance explained, which is invariant upon changes of basis in the space of PWMs. In particular we want to compare the variance explained by the TBA model and models based on a score cutoff. If the log-linear model Eq (5) can explain a significant fraction of the variance in gene expression among genes, then it can be used for this purpose.

**Fig 2:**
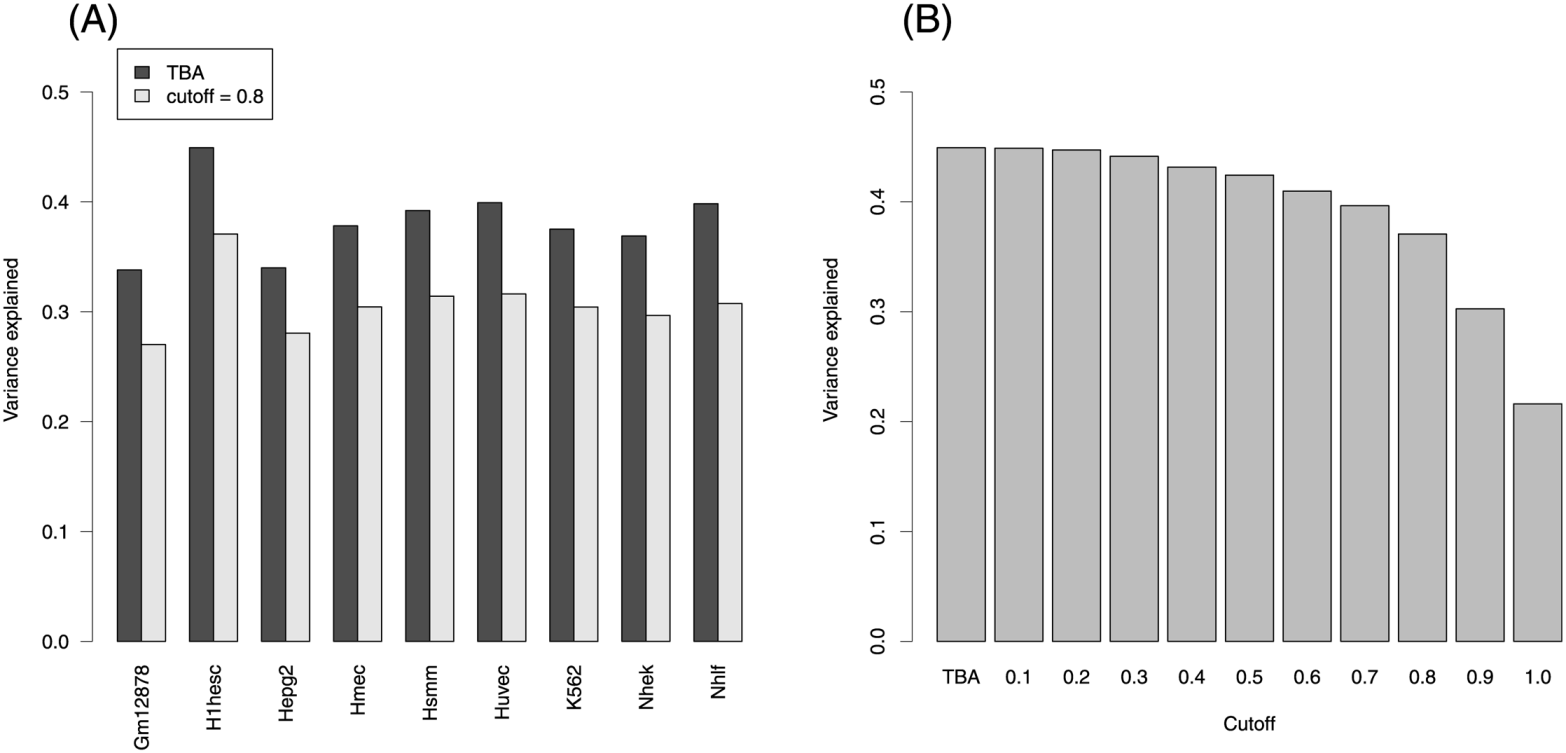
(A) Fraction of gene expression variance explained by models based on TBA and on occupancy with 80% cutoff on the score in nine cell lines. (B) Same as a function of the cutoff for human embryonic stem cells.

To avoid using redundant PWMs describing the same TFs we chose the JASPAR “Core Vertebrate” collection (2009 release) containing 130 PWMs. We considered only genes associated to a single transcript so as to avoid ambiguities in the definition of the Transcription Start Site (TSS). As regulatory region we took 1500 bps upstream and 500 bps downstream of the TSS. Fig 2A compares the fraction of variance explained by the model in each cell line using TBA *vs* occupancy defined with a *C* = 0.8 cutoff, that is 80% of the maximum score. The fraction of variance explained by the TBA model varies between 0.338 and 0.449 (median 0.378). Overfitting is not a serious issue since in a 10-fold cross-validation experiment we obtained a median *R* ^2^ of 0.371. In all cases this is strongly significant, as all P-values are <2.2 ⋅ 10^−16^. For comparison, using MotifLocator with the same PWMs and a 0.8 cutoff and summing over the score of all binding sites produces a median *R* ^2^ of 0.267.

Fig 2A shows that TBA has higher predictive power than occupancy defined at the commonly used 80% cutoff: TBA provides a gain in variance explained between 0.059 and 0.091. These results show that describing regulatory regions in terms of TBA profiles allows to explain a modest but significant part of gene expression, larger than what would be explained by identifying discrete binding sites. Fig 2B shows, using the H1-hESC cell line as an example, that the variance explained is a decreasing function of the cutoff.

We asked how many of the 130 PWMs actually contribute to the prediction of gene expression. Using lasso regression on the same data, with penalization coefficient chosen to minimize cross-validation error, we obtained *R* ^2^ values slightly lower than the full model. The PWMs selected by lasso are in all cases a large subset of the original ones (between 89 and 107 PWMs out of a total of 130), suggesting that the JASPAR Core set has limited redundance. The PWMs selected by lasso regression for the various cell lines are shown in S2 Dataset and S2 Fig.

These data show that TBA profiles are good predictors of both TF binding and gene expression, but we cannot conclude that they predict gene expression through predicting TF binding. Indeed TBA profiles could represent generic sequence features associated to expression levels, largely independent from the ability to bind the specific TFs represented by the PWMs. To gain some insight into this we generated shuffled PWMs which retain the base composition, but not the position specificity, of the original 130 PWMs, and used the TBA for these matrices as regressors of gene expression.

While true PWMs have significantly higher predictive power than shuffled ones (*P* = 0.0039, Wilcoxon paired test), the effect is rather small: shuffled PWMs achive a median *R* ^2^ of 0.353 compared to 0.378 of the original ones. This suggests that much, but not all, the predictive power of TBA profiles is due to their capturing sequence features that are not directly related to the binding sites of the TF they describe. It is an interesting open problem to understand exactly what these features are: indeed these are not trivial, since a model based on base and CpG composition obtained a median *R* ^2^ of 0.248.

### Defining regulatory regions based on chromatin state

It is known that information on chromatin state, or more generally DNA accessibility, can be used to improve the prediction of TF binding and gene expression (see e.g. [26–28]). Histone modification data obtained by ChIP-seq and information on DNA accessibility revealed by DNAse hypersensitivity assays allow the selection of regulatory regions that are able to perform their function by binding trans-acting factors. Therefore we sought a method to incorporate chromatin state information in the TBA model of gene expression.

For the 9 cell lines used above the genome has been classified [16] into 15 chromatin states using a Hidden Markov Models (HMM) based on histone modifications revealed by ChIP-seq experiments. We used this classification to define cell-type-dependent regulatory regions in the following way:

1. we start with the regulatory regions used above, that is 1500 bps upstream and 500 bps downstream of the TSS
2. of these we consider only the parts that are classified into one of the “open” states by [16]
3. if the upstream end of the regulatory regions falls into an open state, we extend the region until the end of the open state; the same extension is performed downstream

**Fig 3:**
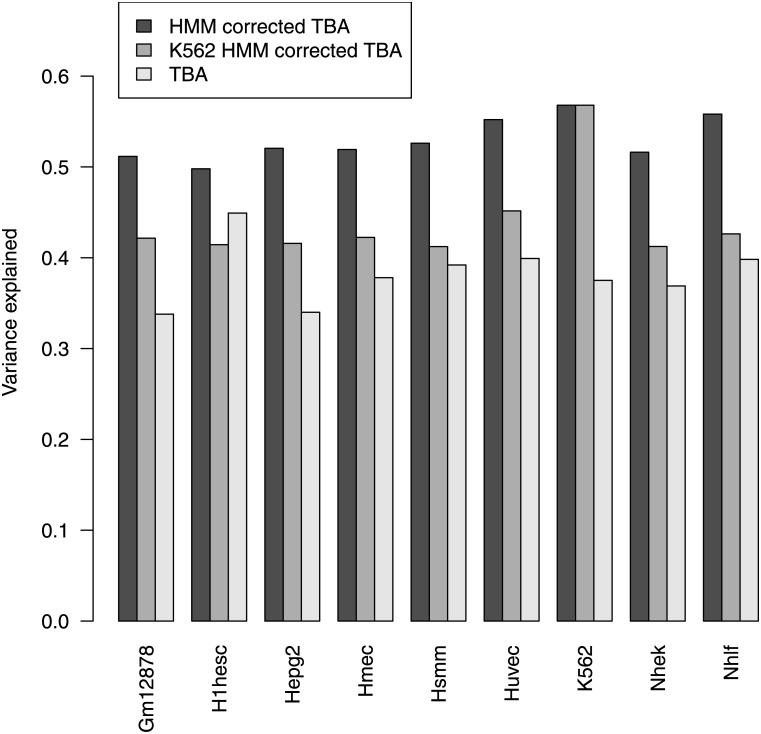
Fraction of gene expression variance explained by models based on chromatin HMM data for each cell line, chromatin HMM data for K562 cells, and TBA alone.

We found that the use of chromatin state information to define regulatory sequences leads to a major improvement in modeling gene expression as shown in Fig 3. The fraction of variance explained by the model is now close to 50%, a remarkable results when keeping in mind that many of the limits discussed below Eq 5 are still in place, such as the use of linear regression and disregarding cooperative effects. Indeed the fraction of variance in gene expression explained by TBA computed on open regions is comparable or higher than what explained in models based on experimental binding data of selected TFs. For example in [26] the authors quote a best adjusted *R* ^2^ of 0.46 for the Gm12878 cells, that we also study, based on a regression model in which the independent variables are histone modifications and ChIP-seq binding data for a set of TFs chosen for their relevance to the cells under investigation. It is noteworthy that a better *R* ^2^ (0.51—see our Fig 3 is obtained by replacing the experimental TF binding data with TBA profiles, which are purely sequence-based (experimental histone modification data are used in both approaches).

Of course here we are not anymore predicting expression from sequence alone, since we are using cell-type-specific chromatin state data. However it is important to keep in mind that the *type* of data needed are not cell-type dependent: the same histone marks were used in the nine cell lines to classify the genome into states. Therefore the approach can be applied to any cellular context without specific knowledge of its biology, such as relevant transcription factors. This marks an important distinction between our approach and works such as [28, 29] in which ChIP-seq data of TFs known to be relevant are used in modeling gene expression.

We asked how important it is to use chromatin state data from the “right” cells in defining the regulatory regions: while chromatin state depends on cell type, it is partially conserved between different cell types [16]. Therefore it is conceivable that using chromatin state information from cells that are similar to the ones under study, or even from any cell, would improve prediction compared to sequence-based predictions. Indeed this is the case, as shown in Fig 3 where we show that the fraction of variance predicted in the 9 cell lines using chromatin state from K562 cells is intermediate between what predicted by TBA alone and using the correct chromatin data (with the notable exception of H1-hESC cells). These data show that chromatin state information greatly improves the prediction of gene expression even when it comes from unrelated cells. This is likely to be due to the fact that, among regulatory states, promoters show the least variability among cell types.

## Discussion

Our goal in studying gene regulation is to be able to predict gene expression based on the sequence and epigenetic modifications of regulatory DNA. In this work we have shown that a description of regulatory sequences in terms of Total Binding Affinity profiles is superior to methods based on the identification of discrete binding sites in predicting TF binding and gene expression. TBA profiles are based on a physical model of TF-DNA interactions [2] which prescribes a specific way of weighing the contribution of binding sites of different affinities.

The relevance of transient binding to weak sites was shown in previous works focusing on yeast [2, 3, 6–10] and Drosophila [1]. Our results suggest that this principle applies to most transcription factors in many different human cell lines.

In particular about half of the variance in expression among genes can be explained using (a) TBA profiles for a set of PWMs describing the binding preferences of ∼130 TFs, (b) a classification of chromatin states derived from histone modification data.

Importantly, we used the same set of PWMs for all cell lines, so that the TBA profiles are independent of the cellular context. Chromatin states are instead context-dependent, but they are derived from a standard set of histone modification data. Therefore no specific knowledge about the biology of each cell line is used in modeling gene expression. In this respect our work differs from several other approaches in which ChIP-seq data for transcription factors known to be relevant in a given context are used to predict expression (see e.g. [26, 28, 29]). Indeed we showed that a model of gene expression based on chromatin state and sequence analysis can perform comparably or better than models using experimental binding data for a manually curated list of relevant TFs.

However it is clear that the predictive power of TBA profiles is not simply due to their ability to predict TF binding. Indeed much, but not all, of such predictive power is retained by shuffled PWMs which are not describing the binding preferences of known TFs. Presumably TBA profiles capture, beside TF binding affinities, other sequence characteristics that are strong predictors of gene expression.

While using chromatin data greatly improves the ability to predict gene expression, using the “correct” chromatin data (that is, of the same cells whose expression we are studying) is not crucial, as a significant improvement is obtained using the same chromatin data for all cell lines. This is important in the perspective of applying this type of modeling to data for which chromatin data for each individual sample are not available (such as large scale expression profiling of cancer [30] or normal individuals [31]).

In this work we have established that the use of TBA leads to increased predictive power when studying both TF binding and gene expression in individual samples. A promising path of further development would be to use TBA profiles to explain changes in gene expression between different samples (especially pathological vs. normal samples) and to infer which transcription factors are involved in such changes, as was done for example in Refs. [32, 33] based on discrete binding sites.

## Supporting Information

S1 Fig Heatmap representing the dependence of the AUC from the score cutoff for 188 PWMs. For each PWM we show the AUC value averaged over all relevant ChIP-seq experiments. These data are the ones used to produce the boxplots of Fig 1.
(PDF)

S2 Fig Lasso regression of gene expression in 9 ENCODE cell lines. Blue: positive coefficient. Red: negative coefficient. White: zero coefficient.
(PDF)

S1 Dataset AUC values. Sheet 1: For each PWM we show the AUC value averaged over all relevant ChIP-seq experiments. Sheet 2: AUCs and P-values of individual PWM/experimet pairs. The P-values are obtained from a Mann-Whitney U test comparing the TBA/occupancy of positive vs negative sequences.
(XLSX)

S2 Dataset Coefficients of lasso regression. Coefficient of each PWM in a lasso regression of gene expression in 9 ENCODE cell lines.
(XLSX)
