# Supplementary material for: Total Binding Affinity Profiles of Regulatory Regions Predict Transcription Factor Binding and Gene Expression in Human Cells
Source: PLoS One. 2015 Nov 24;10(11):e0143627. doi: 10.1371/journal.pone.0143627 (PMC4658012; doi:10.1371/journal.pone.0143627)
Supplement: S1 Fig — For each PWM we show the AUC value averaged over all relevant ChIP-seq experiments. These data are the ones used to produce the boxplots of Fig 1. (PDF) [file pone.0143627.s001.pdf]

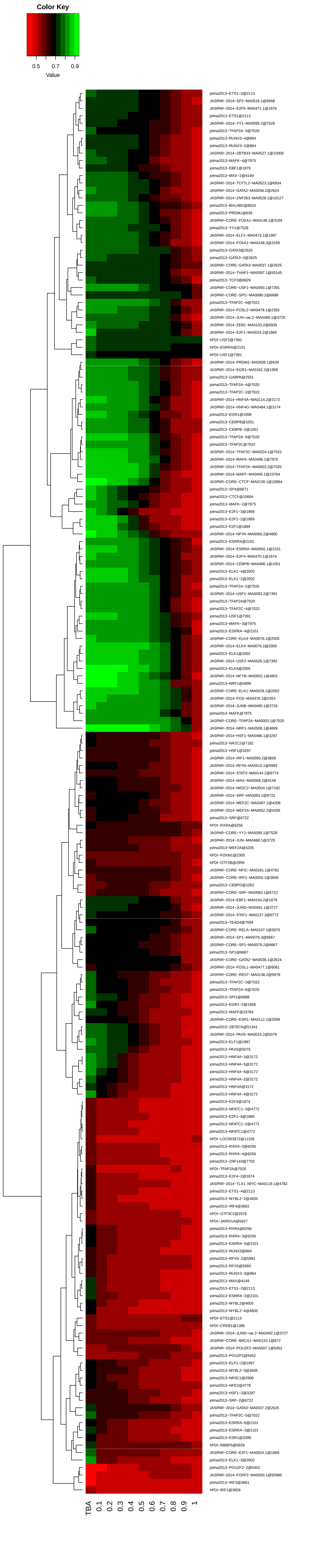

Color Key
0.5
0.7
0.9
Value
jolma2013−ETS1−3@2113
JASPAR−2014−SP2−MA0516.1@6668
JASPAR−2014−E2F6−MA0471.1@1876
jolma2013−ETS1@2113
JASPAR−2014−YY1−MA0095.2@7528
jolma2013−TFAP2A−3@7020
jolma2013−RUNX3−4@864
jolma2013−RUNX3−2@864
JASPAR−2014−ZBTB33−MA0527.1@10009
jolma2013−MAFK−4@7975
jolma2013−EBF1@1879
jolma2013−MAX−2@4149
JASPAR−2014−TCF7L2−MA0523.1@6934
JASPAR−2014−GATA2−MA0036.2@2624
JASPAR−2014−ZNF263−MA0528.1@10127
jolma2013−BHLHB2@8553
jolma2013−PRDM1@639
JASPAR−CORE−FOXA1−MA0148.1@3169
jolma2013−YY1@7528
JASPAR−2014−ELF1−MA0473.1@1997
JASPAR−2014−FOXA1−MA0148.3@3169
jolma2013−GATA3@2625
jolma2013−GATA3−2@2625
JASPAR−CORE−GATA3−MA0037.1@2625
JASPAR−2014−THAP1−MA0597.1@55145
jolma2013−TCF3@6929
JASPAR−CORE−USF1−MA0093.1@7391
JASPAR−CORE−SPI1−MA0080.2@6688
jolma2013−TFAP2C−6@7022
JASPAR−2014−FOSL2−MA0478.1@2355
JASPAR−2014−JUN−var.2−MA0489.1@3725
JASPAR−2014−ZEB1−MA0103.2@6935
JASPAR−2014−E2F1−MA0024.2@1869
hPDI−USF2@7392
hPDI−ESRRA@2101
hPDI−USF1@7391
JASPAR−2014−PRDM1−MA0508.1@639
JASPAR−2014−EGR1−MA0162.2@1958
jolma2013−GABPA@2551
jolma2013−TFAP2A−4@7020
jolma2013−TFAP2C−2@7022
JASPAR−2014−HNF4A−MA0114.2@3172
JASPAR−2014−HNF4G−MA0484.1@3174
jolma2013−EGR1@1958
jolma2013−CEBPB@1051
jolma2013−CEBPB−2@1051
jolma2013−TFAP2A−5@7020
jolma2013−TFAP2C@7022
JASPAR−2014−TFAP2C−MA0524.1@7022
JASPAR−2014−MAFK−MA0496.1@7975
JASPAR−2014−TFAP2A−MA0003.2@7020
JASPAR−2014−MAFF−MA0495.1@23764
JASPAR−CORE−CTCF−MA0139.1@10664
jolma2013−SP4@6671
jolma2013−CTCF@10664
jolma2013−MAFK−2@7975
jolma2013−E2F1−3@1869
jolma2013−E2F1−2@1869
jolma2013−E2F1@1869
JASPAR−2014−NFYA−MA0060.2@4800
jolma2013−ESRRA@2101
JASPAR−2014−ESRRA−MA0592.1@2101
JASPAR−2014−E2F4−MA0470.1@1874
JASPAR−2014−CEBPB−MA0466.1@1051
jolma2013−ELK1−4@2002
jolma2013−ELK1−2@2002
jolma2013−TFAP2A−2@7020
JASPAR−2014−USF1−MA0093.2@7391
jolma2013−TFAP2A@7020
jolma2013−TFAP2C−4@7022
jolma2013−USF1@7391
jolma2013−MAFK−3@7975
jolma2013−ESRRA−4@2101
JASPAR−CORE−ELK4−MA0076.1@2005
JASPAR−2014−ELK4−MA0076.2@2005
jolma2013−ELK1@2002
JASPAR−2014−USF2−MA0526.1@7392
jolma2013−ELK4@2005
JASPAR−2014−NFYB−MA0502.1@4801
jolma2013−NRF1@4899
JASPAR−CORE−ELK1−MA0028.1@2002
JASPAR−2014−FOS−MA0476.1@2353
JASPAR−2014−JUNB−MA0490.1@3726
jolma2013−MAFK@7975
JASPAR−CORE−TFAP2A−MA0003.1@7020
JASPAR−2014−NRF1−MA0506.1@4899
JASPAR−2014−HSF1−MA0486.1@3297
jolma2013−NR2C2@7182
jolma2013−HSF1@3297
JASPAR−2014−IRF1−MA0050.2@3659
JASPAR−2014−RFX5−MA0510.1@5993
JASPAR−2014−STAT3−MA0144.2@6774
JASPAR−2014−MAX−MA0058.2@4149
JASPAR−2014−NR2C2−MA0504.1@7182
JASPAR−2014−SRF−MA0083.2@6722
JASPAR−2014−MEF2C−MA0497.1@4208
JASPAR−2014−MEF2A−MA0052.2@4205
jolma2013−SRF@6722
hPDI−RXRA@6256
JASPAR−CORE−YY1−MA0095.1@7528
JASPAR−2014−JUN−MA0488.1@3725
jolma2013−MEF2A@4205
hPDI−FOXM1@2305
hPDI−GTF2B@2959
JASPAR−CORE−NFIC−MA0161.1@4782
JASPAR−CORE−IRF1−MA0050.1@3659
jolma2013−CEBPD@1052
JASPAR−CORE−SRF−MA0083.1@6722
JASPAR−2014−EBF1−MA0154.2@1879
JASPAR−2014−JUND−MA0491.1@3727
JASPAR−2014−STAT1−MA0137.3@6772
jolma2013−TEAD4@7004
JASPAR−CORE−RELA−MA0107.1@5970
JASPAR−2014−SP1−MA0079.3@6667
JASPAR−CORE−SP1−MA0079.2@6667
jolma2013−SP1@6667
JASPAR−CORE−GATA2−MA0036.1@2624
JASPAR−2014−FOSL1−MA0477.1@8061
JASPAR−CORE−REST−MA0138.2@5978
jolma2013−TFAP2C−3@7022
jolma2013−TFAP2A−6@7020
jolma2013−SPI1@6688
jolma2013−EGR1−2@1958
jolma2013−MAFF@23764
JASPAR−CORE−ESR1−MA0112.2@2099
jolma2013−ZBTB7A@51341
JASPAR−2014−PAX5−MA0014.2@5079
jolma2013−ELF1@1997
jolma2013−PAX5@5079
jolma2013−HNF4A−3@3172
jolma2013−HNF4A−5@3172
jolma2013−HNF4A−6@3172
jolma2013−HNF4A−2@3172
jolma2013−HNF4A@3172
jolma2013−HNF4A−4@3172
jolma2013−E2F4@1874
jolma2013−NFATC1−3@4772
jolma2013−E2F1−4@1869
jolma2013−NFATC1−2@4772
jolma2013−NFATC1@4772
hPDI−LOC653972@11335
jolma2013−RXRA−2@6256
jolma2013−RXRA−4@6256
jolma2013−ZNF143@7702
hPDI−TFAP2A@7020
jolma2013−E2F4−2@1874
JASPAR−2014−TLX1::NFIC−MA0119.1@4782
jolma2013−ETS1−4@2113
jolma2013−MYBL2−2@4605
jolma2013−IRF4@3662
hPDI−GTF3C2@2976
hPDI−JARID1A@5927
jolma2013−RXRA@6256
jolma2013−RXRA−3@6256
jolma2013−ESRRA−5@2101
jolma2013−RUNX3@864
jolma2013−RFX5−2@5993
jolma2013−RFX5@5993
jolma2013−RUNX3−3@864
jolma2013−MAX@4149
jolma2013−ETS1−2@2113
jolma2013−ESRRA−2@2101
jolma2013−MYBL2@4605
jolma2013−MYBL2−4@4605
hPDI−ETS1@2113
hPDI−CREB1@1385
JASPAR−2014−JUND−var.2−MA0492.1@3727
JASPAR−CORE−BRCA1−MA0133.1@672
JASPAR−2014−POU2F2−MA0507.1@5452
jolma2013−POU2F2@5452
jolma2013−ELF1−2@1997
jolma2013−MYBL2−3@4605
jolma2013−NR3C1@2908
jolma2013−NFE2@4778
jolma2013−HSF1−2@3297
jolma2013−SRF−2@6722
JASPAR−2014−GATA3−MA0037.2@2625
jolma2013−TFAP2C−5@7022
jolma2013−ESRRA−6@2101
jolma2013−ESRRA−3@2101
jolma2013−ESR1@2099
hPDI−RBBP5@5929
JASPAR−CORE−E2F1−MA0024.1@1869
jolma2013−ELK1−3@2002
jolma2013−POU2F2−2@5452
JASPAR−2014−FOXP2−MA0593.1@93986
jolma2013−IRF3@3661
hPDI−IRF1@3659
TBA
0.1
0.2
0.3
0.4
0.5
0.6
0.7
0.8
0.9
1
