# Supplementary figures and images for: Total Binding Affinity Profiles of Regulatory Regions Predict Transcription Factor Binding and Gene Expression in Human Cells
Source: PLoS One. 2015 Nov 24;10(11):e0143627. doi: 10.1371/journal.pone.0143627 (PMC4658012; doi:10.1371/journal.pone.0143627)

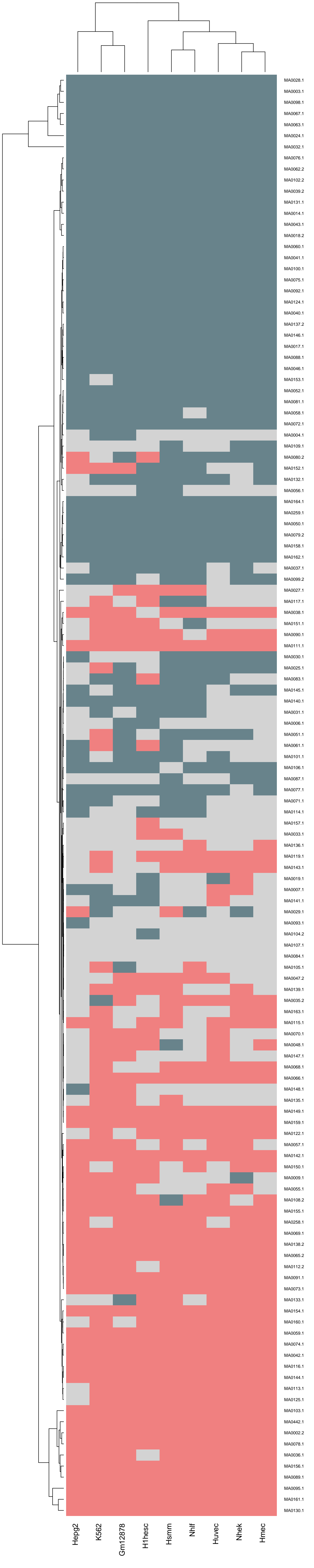

Supplement: S2 Fig — Blue: positive coefficient. Red: negative coefficient. White: zero coefficient. (PDF) [file pone.0143627.s002.pdf]
